# Life-Threatening Microangiopathy or Vitamin Deficiency: A Case Report of the Clinical Manifestations of Pseudo-Thrombotic Microangiopathic Anemia

**DOI:** 10.7759/cureus.20228

**Published:** 2021-12-07

**Authors:** Ian Landry, Tapati Chowdhury, Suemaya Hussein, Leno Thomas

**Affiliations:** 1 Medicine, Icahn School of Medicine at Mt Sinai/NYC Health + Hospitals Queens, Jamaica, USA; 2 Internal Medicine, Icahn School of Medicine, Mount Sinai/NYC Health + Hospitals Queens, Jamaica, USA; 3 Internal Medicine, St George's School of Medicine, True Blue, GRD; 4 Hematology Oncology, Icahn School of Medicine at Mount Sinai, Queens Hospital Center, New York City, USA

**Keywords:** microangiopathic haemolytic anemia, pernicious anemia, acquired ttp, ttp, pseudo-ttp, cobalamin deficiency

## Abstract

Hemolytic anemia with thrombocytopenia and organ damage raises suspicion for thrombotic microangiopathy (TMA), a pathology that results in thrombosis within the small vessels secondary to endothelial injury. While usually attributed to atypical hemolytic uremic syndrome (aHUS) or thrombotic thrombocytopenic purpura (TTP), an increasingly recognized and treatable entity is pseudo-thrombotic microangiopathic anemia (pseudo-TMA) secondary to severe vitamin B-12 deficiency.

While TMA often requires expensive diagnostic testing and can lead to invasive treatment options such as plasma exchange, immunosuppression, and/or complement cascade blocking, pseudo-TMA requires only vitamin supplementation. Therefore, the prompt and accurate diagnosis of this entity is important for the clinician to recognize in order to avoid unnecessary health costs and institute appropriate treatment.

We present the case of a 51-year-old male without any past medical history, who presented with generalized weakness, dyspnea on exertion, and decreased exercise tolerance for several months and was found to have severe microangiopathic anemia with work-up concerning for TTP. After stabilization, he was found to have severe B-12 deficiency secondary to newly diagnosed pernicious anemia and was treated with subcutaneous B-12 injections with improvement in clinical symptoms and laboratory parameters. This presentation highlights the need for prompt diagnosis and high clinical suspicion for vitamin deficiencies as a source of pseudo-microangiopathy.

## Introduction

Thrombotic microangiopathies (TMAs) are a group of rare, yet life-threatening hematologic conditions which are characterized by microangiopathic hemolytic anemia, thrombocytopenia, and microthrombi leading to ischemic tissue injury [1]. While there are subtle differences in their presentation, they share a common pathological process; chiefly, endothelial cell injury leading to vascular damage. The most prevalent manifestations of these disorders are thrombotic thrombocytopenic purpura (TTP) and hemolytic uremic syndrome (HUS). The annual incidence of TTP in adults is estimated to be three cases per 1,000,000 [1], while atypical hemolytic uremic syndrome (aHUS) is estimated to be two cases per 1,000,000 each year in the USA [2]. TTP is caused by severe deficiency of the enzyme ADAMTS13 (ADAM metallopeptidase with thrombospondin type 1 motif 13) or by an inhibitor produced against this enzyme. Physiologically, this enzyme works to regulate blood clotting by cleaving von Willebrand factor multimers, thereby preventing the formation of thrombi within normal circulation [1]. aHUS is caused by a dysregulation of the alternative complement pathway, which leads to increased complement cascade activation [1].

TMAs are largely clinically diagnosed. Thrombocytopenia and anemia with evidence of hemolysis on a peripheral blood smear (i.e., schistocytes) are usually sufficient to make a diagnosis. In TTP, these biochemical signs can be associated with neurologic impairment, kidney failure, and fever, with the full pentad having a mortality of nearly 50% within the first 24 hours [1]. aHUS requires a prompt, clinical diagnosis due to its association with rapidly progressive renal failure. When the need for differentiation between these two entities arises, the activity of ADAMTS13 is measured, with severe deficiency less than 10% being virtually diagnostic [2]. Cobalamin deficiency may result in hematologic characteristics which are clinically similar to TMAs. Ineffective erythropoiesis from cobalamin deficiency results in intramedullary hemolysis, leading to a clinical picture that resembles microangiopathic hemolytic anemia. In patients with significant comorbid conditions such as baseline kidney or liver dysfunction, there can be significant confounding of the diagnostic picture.

## Case presentation

A 51-year old African American male with no significant past medical history presented with generalized weakness, dyspnea on exertion, and decreased exercise tolerance for approximately two months. He also endorsed non-specific vomiting, loss of appetite, and a 30-lb weight loss in the same duration. Notably, he denied any chest pain, cough, hemoptysis, abdominal pain, hematemesis, or melena. He had a recent visit to his home country of Haiti within the past year, but otherwise denied any travel or sick contacts. He stated that he was apparently healthy, working full time, and had not seen a physician in recent years. He immigrated from Haiti as a young man in 1998, is recently divorced, and is employed as a shopkeeper in a grocery store. His social history elicited a significant alcohol use disorder for the past 20 years described as “half a bottle of spirits” every few days. He quit drinking alcohol when his symptoms started two months prior to admission because of intermittent nausea, vomiting, and weight loss, with an inability to eat many of his favorite foods.

On presentation, he was afebrile (98 F), blood pressure was stable at 130/83 mm Hg, with mild tachycardia at 104 bpm, and respiratory rate of 14 breaths per minute with a saturation of 96% on room air. The physical exam was significant only for conjunctival pallor with mild scleral icterus. The patient did not have any neurologic deficits. Laboratory findings (see Table 1) were significant for severe macrocytic anemia with hemoglobin of 4.8 g/dL, hematocrit - 14.6%, mean corpuscular volume - 116.8 fL, high red cell distribution width - 32.2%, and platelet count - 222,000/mcL. A repeat complete blood count after transfusion of two units of packed red blood cells showed pancytopenia with white blood cell count 4,780/mcL, platelets - 110,000/mcL, hematocrit - 19.3%, mean corpuscular volume 107 fL, with an improvement of hemoglobin to 6.4 g/dL. Hepatic function testing showed a mild increase in bilirubin of 1.8 mg/dL and transaminitis with a predominance of aspartate aminotransferase (AST) 230 U/L compared to alanine transaminase (ALT) 141 U/L. Haptoglobin was <20 mg/dL, lactate dehydrogenase >900 U/L. Basic metabolic panel showed normal blood urea nitrogen (14 mg/dL) and creatinine (1.14 mg/dL), ensuring no renal dysfunction. Viral panels including human immunodeficiency (HIV) and hepatitis virus were negative. Serum and urine immunofixation were negative. Thyroid function testing was normal. Quantiferon was positive.

**Table 1:** Trend of significant hematologic laboratory findings

Parameter	Presentation	Day 2 of Admission	3 Weeks After Treatment
Hemoglobin (ref: 14.0-18.0 g/dL)	4.8	6.4	10.4
Hematocrit (ref: 42.0%-52.0%)	14.6%	19.3%	32.5%
Mean Corpuscular Volume (ref: 80.0-99.0 fL)	116.8	107.2	90.3
Platelet Count (ref: 150,000-450,000 mcL)	222,000	110,000	291,000
White Blood Cells (ref: 4.80-10.80 x10(3) /mcL)	6.08	4.78	4.38
Red Cell Distribution Width (ref: 12.0%-15.0%)	32.2%	28.1%	16.3%
Haptoglobin (ref: 34-200 mg/dL)	<20	<20	<20
Lactate Dehydrogenase		>900	296
Total bilirubin (ref: 0.0-1.2 mg/dL)	1.8	1.7	0.4
Serum B-12 level (ref: 232-1245 pg/mL)		<150	407
Reticulocytes (ref: 0.50%-1.50%)		2.14%	0.43%
Schistocytes on PBF (ref: none)	Present, many	Present, many	Absent

Due to the presence of schistocytes on a peripheral smear (up to 7 in some high-power fields), along with severe anemia and thrombocytopenia, prompt hematology consultation was initiated for suspicion of TTP. During the consultation, B-12 level was found to be <150 pg/mL, folate level 5.8 ng/mL (reference: ≥4.7 ng/mL), and direct Coombs’s testing - negative. As the patient had no neurologic or end-organ damage, hematology suggested treating him with intramuscular B-12 for five days to treat non-immune hemolytic anemia in the setting of severe B-12 deficiency. Plasma exchange was not initiated based on the working diagnosis of pseudo-TTP secondary to severe B-12 deficiency. For the sake of completion, ADAMTS13 levels, anti-intrinsic factor antibodies, and anti-parietal cell antibodies were collected. While ADAMTS13 level was pending, blood counts began to improve with 1,000 mcg parenteral B-12 daily supplementations. As the patient’s symptoms improved with stabilization of hemoglobin and hematocrit and with the normalization of platelets, he was confirmed to have pseudo-TTP secondary to severe B-12 deficiency. He was discharged with parenteral cobalamin to complete seven days and continue with oral cobalamin supplementation along with 1 mg of folate oral supplementation daily. On follow-up in the hematology clinic, he was found to be regaining his body weight, had stable hemoglobin, and had a significant improvement in lactate dehydrogenase to 296 U/L. His intrinsic factor antibodies were elevated, indicating pernicious anemia. He was also referred to Pulmonology and commenced rifampin for latent tuberculosis.

## Discussion

TTP is considered a hematologic emergency due to its association with high mortality rates. The treatment of choice for TTP is a very intense form of therapy known as therapeutic plasma exchange, or plasmapheresis, a process in which liquid and solid portions of the blood are separated, treated, then returned to the patient’s circulation. Prompt consideration and proper workup based on broad differentials of microangiopathic hemolytic anemia should include TTP, drug or toxin-induced hemolysis, disseminated intravascular coagulation, malignant hypertension, trauma from prosthetic/mechanical valves, and pre-eclampsia. However, an infrequently considered etiology of anemia, thrombocytopenia, and the presence of schistocytes on peripheral smear includes severe B-12 deficiency. Lack of this vitamin as a cofactor for hematopoiesis can lead to intramedullary hemolysis and insufficient bone marrow production. Previous evaluation by Andres et al. [3] assessing hematologic manifestations or abnormalities in two hundred and one patients with known cobalamin deficiency have shown that while classical findings of anemia and macrocytosis are often seen (37% and 54%, respectively), up to 10% of patients can have life-threatening pseudo-TMAs secondary to cobalamin deficiency. Upon supplementation of cobalamin, nearly 67% of patients with severe hemolytic anemia achieved therapeutic response and resolution of symptoms [3]. Additionally, cobalamin deficiency is often found in patients with certain dietary restrictions, alcohol use disorder, and malabsorption syndromes, all of which are common in the general population.

Population-based studies from the National Health and Nutrition Examination Survey (NHANES) suggest that the general population consumes adequate amounts of vitamin B12, with only 3% of American men and 8% of American women having daily intakes less than the recommended dose [4]. Pernicious anemia is a leading cause of cobalamin deficiency in the developed world. As an irreversible autoimmune disease, antibodies are produced against the parietal cells of the stomach and lead to failure to produce intrinsic factor, the necessary co-factor for the binding and absorption of cobalamin. Noel et al. [5] compared patients with pseudo-TMA to patients with TTP and found that pseudo-TMA patients had higher median lactate dehydrogenase (LDH) levels, lower reticulocyte counts, and higher platelet counts. Similar evaluations by Chen et al. [6] further subdivided patients with pseudo-TMA secondary to pernicious anemia and studied their common characteristics. They found that 61.5% (eight of 13) of the current patients identified in the literature had severely elevated levels of LDH. The authors concluded that patients with features of macrocytosis, elevated LDH, and hyperbilirubinemia in the setting of the cytopenias should raise concern for pernicious anemia-induced pseudo-TMA.

## Conclusions

The recognition of serious adverse effects not classically associated with severe cobalamin deficiency can lead to inappropriate treatment and invasive procedures which unduly burden both patient and healthcare system. While our report is not unique, it does supplement previous findings of pseudo-TMAs and emphasizes the need for prompt diagnosis and treatment. Pseudo-TTP should always be in the differential of a patient with signs of hemolytic anemia, thrombocytopenia, and schistocytes on peripheral smear. Patients who present with abnormal dietary history, a social history of a significant alcohol use disorder, or evidence of personal or family history of auto-immune diseases should raise a high suspicion of pernicious anemia. Additionally, the findings of macrocytosis elevated LDH, and low reticulocyte count may also assist in the diagnostic dilemma. In patients who have signs of multiple risk factors for vitamin B-12 deficiency, supplementation should be considered at the onset of presentation as it can have a rapid improvement in bone marrow production and normalization of cell parameters. Additionally, early consideration and supplementation with B-12 can avoid plasmapheresis, a costly and invasive therapy that will ultimately be an ineffective treatment for pseudo-TTP.
